# Rikkunshito, a ghrelin potentiator, ameliorates anorexia–cachexia syndrome

**DOI:** 10.3389/fphar.2014.00271

**Published:** 2014-12-10

**Authors:** Naoki Fujitsuka, Yasuhito Uezono

**Affiliations:** ^1^Tsumura Research Laboratories, Tsumura & Co.Ibaraki, Japan; ^2^Division of Cancer Pathophysiology, National Cancer Center Research InstituteTokyo, Japan

**Keywords:** rikkunshito, ghrelin, anorexia, cachexia, weight loss

## Abstract

Anorexia–cachexia syndrome develops during the advanced stages of various chronic diseases in which patients exhibit a decreased food intake, weight loss, and muscle tissue wasting. For these patients, this syndrome is a critical problem leading to an increased rate of morbidity and mortality. The present pharmacological therapies for treating anorexia–cachexia have limited effectiveness. The Japanese herbal medicine rikkunshito is often prescribed for the treatment of anorexia and upper gastrointestinal (GI) disorders. Thus, rikkunshito is expected to be beneficial for the treatment of patients with anorexia–cachexia syndrome. In this review, we summarize the effects of rikkunshito and its mechanisms of action on anorexia–cachexia. Persistent loss of appetite leads to a progressive depletion of body energy stores, which is frequently associated with cachexia. Consequently, regulating appetite and energy homeostasis is critically important for treating cachexia. Ghrelin is mainly secreted from the stomach, and it plays an important role in initiating feeding, controlling GI motility, and regulating energy expenditure. Recent clinical and basic science studies have demonstrated that the critical mechanism of rikkunshito underlies endogenous ghrelin activity. Interestingly, several components of rikkunshito target multiple gastric and central sites, and regulate the secretion, receptor sensitization, and degradation of ghrelin. Rikkunshito is effective for the treatment of anorexia, body weight loss, muscle wasting, and anxiety-related behavior. Furthermore, treatment with rikkunshito was observed to prolong survival in an animal model of cachexia. The use of a potentiator of ghrelin signaling, such as rikkunshito, may represent a novel approach for the treatment of anorexia–cachexia syndrome.

## INTRODUCTION

Anorexia–cachexia syndrome is characterized by decreased food intake, hypoalbuminemia, weight loss, and muscle tissue wasting (Tan et al., 2014). This syndrome is observed in patients with advanced stages of various chronic diseases (von Haehling and Anker, 2010), and is a cause of their increased rate of morbidity and mortality (Evans et al., 2008). The treatment of anorexia–cachexia is, therefore, critically important for improving quality of life (QOL) in patients. The onset and development of anorexia–cachexia syndrome is typically associated with an increase in pro-inflammatory cytokine levels (Plata-Salaman, 2000). Therefore, megestrol acetate (Ruiz Garcia et al., 2013) and glucocorticoids are options for the pharmacological therapy of anorexia–cachexia; however, they have limited efficacy (Nelson, 2000; Jatoi et al., 2002). Recently, ghrelin, because of its orexigenic activity, has been suggested as beneficial to treat anorexia–cachexia syndrome (Molfino et al., 2014). Ghrelin is involved in eliciting feeding, controlling gastrointestinal (GI) motility, and regulating energy expenditure and body weight. Thus, clinical trials of ghrelin analogs in cancer cachexia are ongoing (Currow and Abernethy, 2014; Pietra et al., 2014).

Kampo medicine is Japanese traditional herbal medicine standardized with respect to the quality and quantity of ingredients under the Japanese Ministry of Health, Labour, and Welfare. It has been developed through clinical and laboratory studies based on Western-adopted experiment-based approaches (Yu et al., 2006). Rikkunshito, a type of Kampo medicine, is widely prescribed as a remedy for various upper GI syndromes. The adverse drug reaction reports involve hepatobiliary disorders, pseudoaldosteronism, and myopathy. Rikkunshito is manufactured by spray-drying a hot water extract of a mixture of eight varieties of the following crude drugs: *Atractylodis lanceae rhizoma* (4.0 g), *Ginseng radix* (4.0 g), *Pinelliae tuber* (4.0 g), *Poria* (4.0 g), *Zizyphi fructus* (2.0 g), *Aurantii nobilis pericarpium* (2.0 g), *Glycyrrhizae radix* (1.0 g), and *Zingiberis rhizoma* (0.5 g). There is increasing scientific evidence supporting the clinical use of rikkunshito (Takeda et al., 2012). It has been demonstrated that rikkunshito improves anorexia and cachexia, and the improvement is mediated by promoting endogenous ghrelin activity (Suzuki et al., 2012). A better understanding of rikkunshito’s mechanism of action and its active components will contribute to the development of new therapies to improve QOL and potentially to prolong survival in patients with anorexia–cachexia syndrome. The present article reviews the pharmacological effects and clinical benefits of rikkunshito in anorexia–cachexia syndrome.

## CLINICAL APPLICATIONS OF RIKKUNSHITO FOR GI DISORDERS

Functional dyspepsia, which is classified as a functional GI disorder (FGID), is defined as a disease with dyspeptic symptoms, such as postprandial fullness, early satiety, and epigastric burning, and there is no evidence of a structural disease that is likely to explain the symptoms (Tack et al., 2006). Patients with functional dyspepsia exhibit gastric dysmotility, such as delayed gastric emptying (Stanghellini et al., 1996) and impaired gastric accommodation (Tack et al., 1998).

Several clinical studies have demonstrated the effectiveness of rikkunshito in the treatment of GI disorders, including anorexia and gastric dysmotility. Tatsuta and Iishi (1993) reported that the administering rikkunshito, which is named Liu-Jun-Zi-Tang in China, for 7 days accelerated gastric emptying and reduced GI symptoms in 22 patients with chronic idiopathic dyspepsia. Placebo treatment, administered to 20 patients, produced no significant effects (Tatsuta and Iishi, 1993). A large-scale comparative clinical study of 235 patients with dysmotility-like dyspepsia was conducted (Harasawa et al., 1998). Rikkunshito-treated patients (*n* = 118) were given 2.5 g of rikkunshito three times a day for 2 weeks, and placebo-treated patients (*n* = 117) were given 2.5 g of placebo, including 2.5% rikkunshito, as control. As a result, the dysmotility-like dyspepsia generalized improvement rate (DDGI) was significantly higher in the rikkunshito-treated group than in the placebo group. Moreover, rikkunshito was effective in improving anorexia in patients with severe or moderate dyspeptic symptoms. Recently, a multicenter, randomized, placebo-controlled, parallel-group trial of rikkunshito in 247 patients with functional dyspepsia was conducted (Suzuki et al., 2014). The administration of 2.5 g of rikkunshito three times a day for 8 weeks reduced dyspepsia; epigastric pain was significantly improved and postprandial fullness tended to improve compared to the placebo treatment group. There were no severe adverse events in either group.

Gastroesophageal reflux disease (GERD) is often associated with decreased upper GI motility. The therapeutic effects of rikkunshito were reported in proton pump inhibitor (PPI)-refractory patients with GERD or non-erosive reflux disease (NERD). Four-week treatment with rikkunshito (7.5 g/day) in combination with the PPI rabeprazole (RPZ) significantly decreased the frequency scale for the symptoms of GERD (FSSG score) in 104 patients with GERD, which is similar to the decrease observed in response to treatment with a double dose of RPZ (Tominaga et al., 2012). In a randomized, placebo-controlled, double-blind clinical trial for 242 patients with PPI-refractory NERD, treatment for 4 or 8 weeks with rikkunshito (7.5 g/day) improved their mental component summary (MCS) scores in the Short-Form Health Survey-8 (SF-8), which was especially more effective in patients with a low body mass index (<22). Moreover, rikkunshito significantly improved the acid-related dysmotility symptoms of FSSG in female and elderly patients (≥65 years; Sakata et al., 2014; Tominaga et al., 2014).

Additionally, several clinical reports have provided evidence for the therapeutic effects of rikkunshito on GI symptoms and function (Kusunoki et al., 2010; Morita et al., 2012; Gunji et al., 2013; Tokashiki et al., 2013; Uehara et al., 2013).

## BASIC STUDIES OF RIKKUNSHITO ON ANOREXIA AND GI DYSFUNCTION

Physical or psychological stress can cause anorexia and functional disorders in the upper GI tract. Several basic studies of rikkunshito on stress-related anorexia in animals have been reported. Saegusa et al. (2011) constructed a stress model by transferring mice from group-housed cages to individual cages, which are novel environments for mice. The mice stressed by the novel environment exhibited a decrease in food intake 1 and 3 h after stress, which was suppressed by pre-treatment with rikkunshito (500 mg/kg, p.o.) 1 h before the stress (Saegusa et al., 2011). Various psychological factors contribute to decreased food intake among the elderly population. Nahata et al. (2013) reported that exposure of aged mice (79–80 weeks old) to a novel environment markedly decreased food intake compared with that of young mice (6 weeks old). Rikkunshito (1000 mg/kg, p.o.) administration attenuated the decrease in 24-h food intake in stressed aged mice (Nahata et al., 2013).

Urocortin 1 (UCN), a stress hormone, acts on corticotropin-releasing factor (CRF) receptors in the brain and induces anorexia. Yakabi et al. (2011) reported that rikkunshito (1000 mg/kg, p.o.) restored the reduction of food intake in rats with intracerebroventricular administration of UCN (300 pmol). Additionally, the following studies demonstrated that the alpha-2 adrenergic receptor pathway contributes to the associated reduction in food intake (Yakabi et al., 2014).

The efficacy of rikkunshito in the treatment of GI disorders was observed in patients with dysmotility-like dyspepsia (Harasawa et al., 1998). Nahata et al. (2014) also reported the effect of rikkunshito on gastric function in an acute restraint stress mouse model. Mice exposed to restraint stress for 60 min exhibited delayed gastric emptying. Gastric motility, which was wirelessly measured using a strain gage force transducer, was also decreased by restraint stress. Rikkunshito (250 mg/kg, p.o.) administration improved the restraint stress-induced delayed gastric emptying and decreased postprandial gastric contractions (Nahata et al., 2014). These findings suggest that rikkunshito ameliorates several types of stress-induced anorexia and gastric dysmotility.

## RIKKUNSHITO’S MECHANISM OF ACTION

### GHRELIN

Ghrelin is a 28-amino-acid peptide that is mainly secreted from the X/A-like cells in the stomach, and several tissues, including the brain, have small levels of ghrelin. It acts as a natural ligand for the growth hormone secretagogue receptor (GHS-R). Acylation of Ser-3 by the addition of *n*-octanoic acid via the polytopic membrane-bound enzyme ghrelin *O*-acyltransferase (GOAT) is essential for the biological activity of ghrelin via the GHS-R (Kojima et al., 1999; Gnanapavan et al., 2002; Yang et al., 2008).

Ghrelin plays role not only in growth hormone secretion (Kojima et al., 1999) but also in initiating feeding as an appetite stimulant (Nakazato et al., 2001). The plasma ghrelin levels increase in response to prolonged fasting and they decrease rapidly after feeding, suggesting that peripheral ghrelin is significant for appetite regulation (Cummings et al., 2001; Tschop et al., 2001). Ghrelin signals are transmitted to the *nuclei* of the solitary tract via the vagal afferent pathway and they activate the orexigenic neuropeptides neuropeptide Y (NPY) and agouti-related peptide (AgRP) neurons in the hypothalamic arcuate nucleus (ARC), resulting in appetite stimulation (Date et al., 2002; Chen et al., 2004). Additionally, ghrelin has much broader physiologic functions (Kojima and Kangawa, 2005), including controlling GI motility (Fujino et al., 2003), regulating energy expenditure (Asakawa et al., 2001), and suppressing inflammation (Dixit et al., 2004; Granado et al., 2005).

The central or peripheral administration of ghrelin strongly stimulates food intake and increases fat mass, leading to weight gain in animals (Tschop et al., 2000; Asakawa et al., 2001; Nakazato et al., 2001). The intravenous administration of ghrelin in healthy humans increased visual analog scores for appetite and energy intake from a buffet lunch by 28% (Wren et al., 2001). These results suggest the possible clinical applications of ghrelin as a potent stimulator of appetite.

### PROMOTION OF GHRELIN ACTIVITY BY RIKKUNSHITO

The inhibitory effects of rikkunshito on anorexia and gastric dysmotility are thought to be involved in promoting endogenous ghrelin activity. Takeda et al. (2008) demonstrated that rikkunshito ameliorated anorexia in rats treated with cisplatin by inhibiting the decrease of ghrelin levels in the plasma. This is the first report showing that rikkunshito stimulates ghrelin secretion in rats (Takeda et al., 2008). Selective serotonin reuptake inhibitors (SSRIs), including fenfluramine, decreased the plasma ghrelin levels and changed GI motilities in rats. The oral administration of rikkunshito to fenfluramine-treated rats increased the plasma ghrelin levels, food intake, and gastric emptying rate and improved GI dysmotility. The positive effects of rikkunshito on dyspeptic symptoms disappeared after treatment with the GHS-R antagonist (D-Lys3)-GHRP-6, suggesting it mediates the ghrelin signal (Fujitsuka et al., 2009). The ghrelin-mediated appetite-stimulatory effect of rikkunshito was also observed in novel-environment-stressed mice (Saegusa et al., 2011) and UCN-treated rats (Yakabi et al., 2011). Intra-gastric administration of rikkunshito (4 g) is reported to induce fasted phasic contractions in the duodenum and jejunum and to accelerate gastric emptying in dogs. The plasma ghrelin level 150 min after the administration of rikkunshito was significantly higher than the control value (Yanai et al., 2013). Wang et al. (2014) reported that rikkunshito enhanced the fasting plasma levels of ghrelin and alleviated the delayed gastric empty in L-dopa/carbidopa-treated naïve and Parkinson’s disease rats, partially through ghrelin-related mechanisms.

Ghrelin is predominantly produced in gastric X/A-like cells and activates the orexigenic neuropeptides NPY/AgRP in the hypothalamus through the GHS-R in the vagal afferent terminal in the stomach (Date et al., 2002). Rikkunshito-treated rats exhibited elevated gene expression of gastric ghrelin and hypothalamic NPY. The afferent activity of the gastric vagus nerve decreased with the intravenous administration of ghrelin. A similar effect was observed with the intraduodenal administration of rikkunshito (1,000 mg/kg; Asakawa et al., 2001; Fujitsuka et al., 2011). Gastric ghrelin signals induced by the administration of ghrelin (10 ng, i.v.) or rikkunshito (1,000 mg/kg, i.d.) stimulated the efferent activities of both the gastric and the celiac branches of the vagus nerve, which is involved in GI motor activities (Fujino et al., 2003). These findings suggest that rikkunshito activates the ghrelin signal in the vagus nerve. Additionally, gastric vagotomy eliminated the stimulatory effect of ghrelin (10 ng, i.v.) on the efferent activities of the gastric vagus nerve but did not influence the effects of rikkunshito (1,000 mg/kg, i.d.), suggesting rikkunshito acts in part through the GHS-R in the hypothalamus.

Clinical trials have revealed a significant increase in the concentration of circulating ghrelin with rikkunshito. Matsumura et al. (2010) demonstrated that the administration of rikkunshito (7.5 g per day) for 2 weeks increased the plasma ghrelin levels in 21 healthy volunteers. Takiguchi et al. (2013) demonstrated a significant attenuation of GI symptoms after treatment with 2.5 g of rikkunshito for 4 weeks in 25 patients who had undergone gastrectomy. The mean ratio of the acyl-/total ghrelin concentration increased after rikkunshito administration (Takiguchi et al., 2013). Arai et al. (2012) conducted a parallel, randomized, controlled trial of rikkunshito or domperidone for 4 weeks for 27 patients with functional dyspepsia. Upper GI symptoms based on the Gastrointestinal Symptom Rating Scale (GSRS) score were ameliorated in both groups, but the efficacy of rikkunshito was accompanied by an increase in the ghrelin levels (Arai et al., 2012).

## TARGET MOLECULES AND ACTIVE COMPONENTS OF RIKKUNSHITO

**FIGURE 1:**
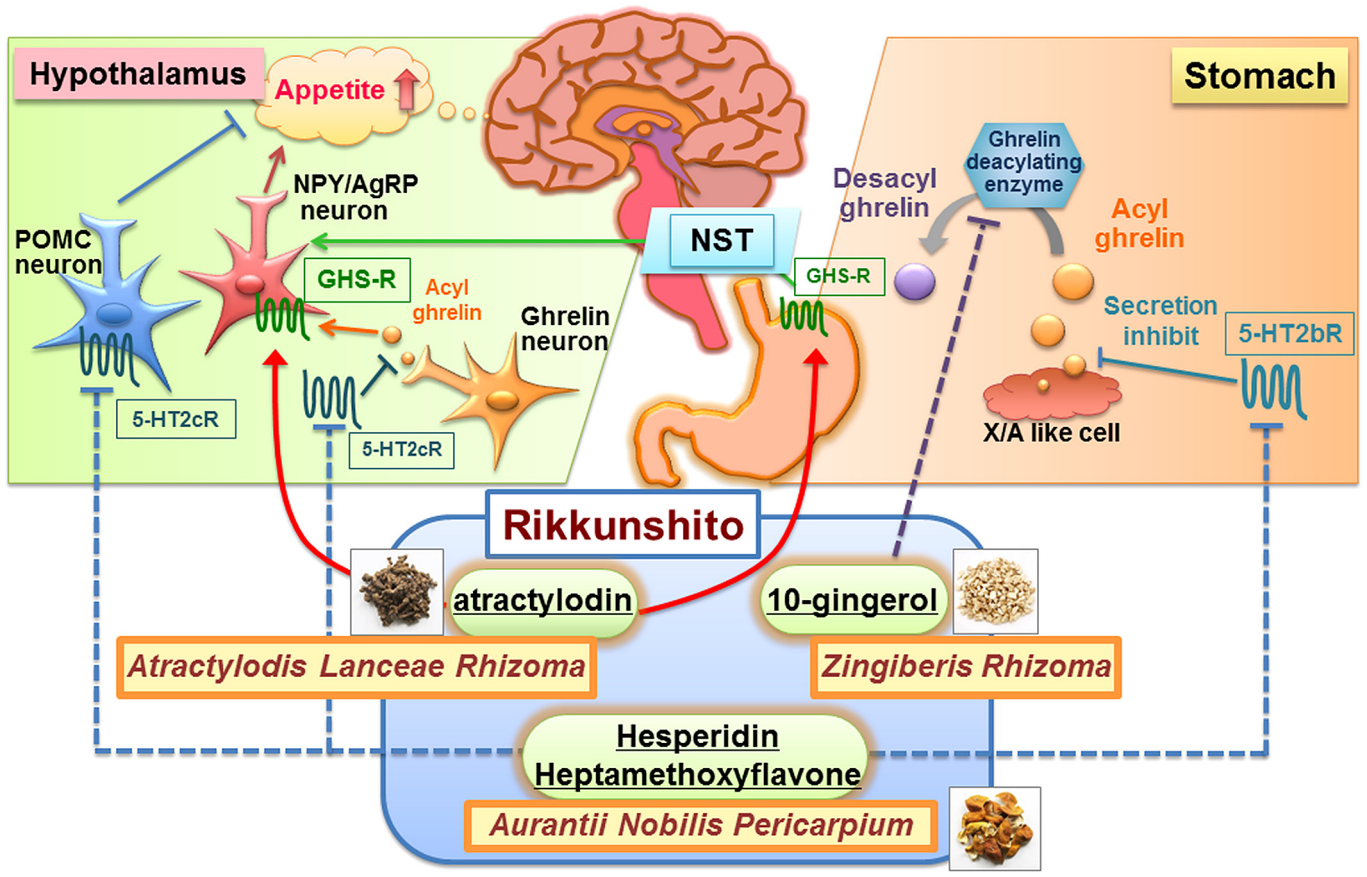
**Summary of the mechanism of action by some ingredients of rikkunshito.** Rikkunshito synergistically promotes endogenous ghrelin activity. The ingredients of rikkunshito such as heptamethoxyflavone and hesperetin (an aglycon form of hesperidin), antagonize 5-HT2b/2c receptors in the stomach and the hypothalamus, which are related to ghrelin secretion. Atractylodin activates ghrelin receptors in the stomach and the hypothalamus. 10-Gingerol inhibits ghrelin deacylating enzymes in various tissues, including the stomach. The potentiation of ghrelin signaling pathways may be responsible for rikkunshito’s attenuation of anorexia. POMC, pro-opiomelanocortin; GHS-R, ghrelin receptor; NST, nucleus tractus solitarii; NPY, neuropeptide Y; AgRP, agouti-related peptide.

Rikkunshito was reported to regulate ghrelin secretion, ghrelin receptor sensitization, and ghrelin degradation, suggesting that rikkunshito synergistically promotes endogenous ghrelin activity (Uezono et al., 2012). As shown **Table 1**, some target molecules and active components of rikkunshito involved in these effects were identified. Summary of the rikkunshito’s mechanism of action was shown **Figure 1**.

**Table 1:** Target molecules and active components of rikkunshito.

Target molecules	Active components (Crude drug)	Structure	Reference
5-HT2b/2cR	3,3,4,5,6,7,8- Heptamethoxyfkvone (Aurantii nobilis pericarpium)	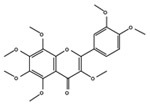	Takeda et al. (2008)
	
	Hesperetin an aglycon form of hesperidin (Aurantii nobilis pericarpium)	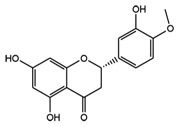	Takeda et al. (2008)
	
	Isoliquiritigenin (Glycyrrhizae radix)	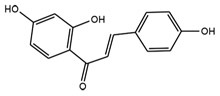	Takeda et al. (2008)
	
GHS-R	Atractylodin (Atractylodis lanceae rhizoma)	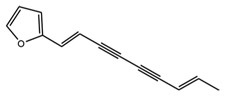	Fujitsuka et al. (2011)
	
Ghrelin deacylating enzymes	10-Gingerol (Zingiberis rhizoma)	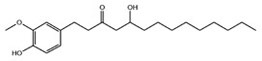	Sadakane et al. (2011)

### SEROTONIN 2b/2c RECEPTORS

The central serotonin (5-HT) system is implicated in the processes of meal satiation and satiety (Vickers et al., 2001, 2003; Halford et al., 2007). Takeda et al. (2008) demonstrated that 5-HT produced during treatment with cisplatin stimulates the 5-HT2b receptor in the stomach and the 5-HT2c receptor in the central nervous system, resulting in decreased plasma ghrelin. Heptamethoxyflavone, hesperetin (an aglycon form of hesperidin), and isoliquiritigenin, which are components of rikkunshito, antagonize 5-HT2b/2c receptors and stimulate ghrelin secretion in cisplatin-treated rats. Fenfluramine decreased plasma ghrelin and changed ghrelin-mediated GI motor activities through the central 5-HT2c receptor. The oral administration of hesperidin to fenfluramine-treated rats restored GI dysmotility (Fujitsuka et al., 2009). These findings suggest that these components of rikkunshito augment ghrelin secretion through antagonizing the 5-HT2b/c receptors.

Nahata et al. (2013) demonstrated that exposure of aged mice to a novel environment up-regulated hypothalamic 5-HT2c receptor mRNA expression. 5-HT2c receptor signaling enhancement and the subsequent activation of the CRF-corticosterone pathway were involved in novelty-induced hypophagia in aged mice. The 5-HT2c receptor antagonist SB242084 or rikkunshito administration attenuated the decrease in food intake and increased corticosterone levels in stressed aged mice (Nahata et al., 2013).

Additionally, *in vitro* studies using fura-2 microfluorometry have revealed that rikkunshito influences the effect of 5-HT on hypothalamic neurons. Administration of 10^-5^ mol/L 5-HT increased the cytosolic Ca^2+^ concentration in single neurons isolated from the paraventricular nucleus (PVN) of rats. These changes were inhibited by the administration of 100 μg/mL of rikkunshito to the PVN neurons, 83% of which subsequently demonstrated immunoreactivity to CRF (Fujitsuka et al., 2011). Administration of 5-HT increased the cytosolic Ca^2+^ concentration in ARC neurons, and 80% of the 5-HT-responsive neurons were immunoreactive to pro-opiomelanocortin (POMC). Rikkunshito and isoliquiritigenin counteracted 5-HT-induced 5-HT2c receptor-mediated Ca^2+^ signaling in POMC neurons (Arai et al., 2013). These results suggest that the inhibition of the 5-HT 2c receptor expressed on CRF neurons (Heisler et al., 2007) or POMC neurons (Heisler et al., 2003) could be responsible for rikkunshito’s attenuation of anorexia.

### GHRELIN RECEPTOR

Growth hormone secretagogue receptors are located in peripheral several tissues and central neurons, including NPY neurons. Ghrelin increases the cytosolic Ca^2+^ concentration in the NPY neurons of the hypothalamic ARC (Kohno et al., 2003), and this effect is linked to stimulation of appetite (Kohno et al., 2007). Compared to 10^-12^ mol/L ghrelin administration, pretreatment with rikkunshito enhanced the ghrelin-induced increase in cytosolic Ca^2+^ levels in isolated fura-2-loaded rat ARC neurons, which were subsequently shown to be NPY neurons by immunocytochemistry (Fujitsuka et al., 2011). Furthermore, Ca^2+^ imaging analysis using fluorescence of G-CAMP2 revealed that rikkunshito (100 μg/mL) had no effect on the cytosolic Ca^2+^ concentration; however, it enhanced the duration of the cytosolic Ca^2+^ concentration increased by 10^-7^ mol/L ghrelin in GHS-R-expressing COS cells. Rikkunshito also increased the binding activity of [^125^I]-ghrelin to the GHS-R.

To identify active component of rikkunshito, the 43 compounds (100 μmol/L) contained in rikkunshito were screened. As a result, atractylodin showed a marked increase in ghrelin/GHS-R binding activity. Atractylodin also sustained the ghrelin-induced cytosolic Ca^2+^ increase in GHS-R-expressing cells (Fujitsuka et al., 2011). These results suggest that atractylodin is active component of rikkunshito, which potentiates the action of ghrelin by presumably sensitizing the ghrelin receptor.

Nahata et al. (2012) demonstrated that ghrelin increased antral motility in sham-operated rats but not in GERD rats. However, in GERD rats treated with rikkunshito, a significant increase in antral motility by ghrelin was observed (Nahata et al., 2012). These findings suggest that the physiological functions of endogenous ghrelin are potentiated by rikkunshito acting on GHS-R signaling, which may be mediated by atractylodin, an active component of rikkunshito.

### GHRELIN DEGRADING ENZYME

Sadakane et al. (2011) reported that rikkunshito increased the acyl- to desacyl-ghrelin (A/D) ratio in plasma from cisplatin-treated rats. Several components of rikkunshito have inhibitory activities against ghrelin deacylating enzymes. 10-gingerol, an active component of rikkunshito, inhibited exogenous ghrelin deacylation in rats. These results suggest that the increase in the plasma ghrelin level by rikkunshito is mediated, at least in part, through inhibiting the ghrelin degrading enzyme (Sadakane et al., 2011).

## CACHEXIA

### PATHOGENESIS OF ANOREXIA–CACHEXIA SYNDROME

Anorexia–cachexia syndrome develops during the advanced stages of various chronic diseases, such as malignant cancer, chronic heart failure, chronic kidney disease, and chronic obstructive pulmonary disease (von Haehling and Anker, 2010). This syndrome results in a decreased QOL and increased morbidity and mortality. Cachexia is diagnosed by the presence of weight loss exceeding 5% within the previous 3–12 months, anorexia, loss of skeletal muscle, and biochemical abnormalities, such as increased inflammatory markers, anemia, and hypoalbuminemia (Evans et al., 2008). In particular, anorexia is very important in the diagnosis and treatment of cachexia-associated weight loss because a persistent loss of appetite leads to a progressive depletion of body energy stores (Argiles et al., 2010).

Cytokines participate in the development and/or progression of anorexia–cachexia (Plata-Salaman, 2000). Cancer cachectic animals exhibit increased plasma levels of cytokines, such as interleukin-1β (IL-1β), interleukin-6 (IL-6), tumor necrosis factor-α (TNF-α), and leukemia inhibitory factor (LIF), which are either produced by cancer cells or released by the host immune system in response to the cancer (Mori et al., 1991; Inui, 2002; Ebrahimi et al., 2004; Perboni and Inui, 2006; Tisdale, 2009). These cytokines in the brain or circulation augment the release of anorexigenic hormones, including 5-HT, leptin, cholecystokinin (CCK), peptides derived from the glucagon precursor, and insulin (Shintani et al., 1993; Laviano et al., 2000). Increased 5-HT concentration in the hypothalamus is demonstrated in animals with cancer (Wang et al., 2003). Megestrol acetate and glucocorticoids are options for the pharmacological therapy of anorexia–cachexia, but they have limited effectiveness (Nelson, 2000; Jatoi et al., 2002).

### ROLE OF GHRELIN ON CACHEXIA

Circulating ghrelin levels are reported to increase in underweight patients with malignancy-associated cachexia (Shimizu et al., 2003; Garcia et al., 2005) and tumor-bearing animals (Terawaki et al., 2014), suggesting a failure of the adaptive feeding response by endogenous ghrelin (Schwartz et al., 1995; Schwartz and Seeley, 1997; Flier, 1998). The plasma ghrelin levels were higher in tumor-bearing rats than in free-fed normal rats, but they were significantly lower than in pair-fed normal rats. Decreases in the hypothalamic expression of NPY and AgRP were also observed in tumor-bearing rats compared to pair-fed controls. Therefore, cancer anorexia–cachexia is characterized as a decrease in ghrelin signaling with both ghrelin insufficiency and resistance, which is mediated by excessive hypothalamic interactions of 5-HT and CRF through the 5-HT2c receptor (Fujitsuka et al., 2011). Administration of ghrelin (Hanada et al., 2003) or GHS-R agonist (Currow and Abernethy, 2014; Pietra et al., 2014) can overcome resistance to the appetite-stimulating effects of the endogenous ghrelin and improve food intake and weight gain in human and animal subjects with cachexia.

Additionally, ghrelin inhibits the production of anorectic proinflammatory cytokines, including IL-1β, IL-6, and TNF-α (Dixit et al., 2004). DeBoer et al. (2007) demonstrated that ghrelin-treated animals with cancer cachexia have a significant increase in the expression of AgRP and NPY with decreased expression of the IL-1 receptor-I transcript in the hypothalamus. Chronic kidney disease is associated with an increase in inflammatory cytokines, resulting in cachexia with muscle loss. Ghrelin-treated nephrectomized animals had a decrease in circulating inflammatory cytokines and IL-1 receptor expression in the brainstem. Ghrelin treatment in uremia results in improved lean mass accrual, which is in part due to suppressed muscle proteolysis and possibly related to anti-inflammatory effects (Deboer et al., 2008; Suzuki et al., 2013). Ghrelin administration reduced lung inflammation, protected alveolar epithelial cells, and ameliorated lung fibrosis in a bleomycin (BLM)-induced acute lung injury model in mice (Imazu et al., 2011). The combination of orexigenic and anti-inflammatory actions suggests that ghrelin has benefits in the treatment of cachexia.

### ANTI-CACHECTIC EFFECT OF RIKKUNSHITO

Increasing evidence from experimental animal models has shown that rikkunshito, which synergistically promotes endogenous ghrelin activity, ameliorates several types of cachexia. These findings suggest that rikkunshito may be more effective for ghrelin resistance such as cancer anorexia–cachexia than treatment of ghrelin or GHS-R agonists.

Rikkunshito improved anorexia, gastrointesitinal dysmotility, muscle wasting, and anxiety-related behavior in AH-130 hepatoma-bearing rats (Fujitsuka et al., 2011). The authors observed anorexia 5 days after intraperitoneal injection of tumor in rats, but the administration of rikkunshito (1000 mg/kg, p.o.) increased food intake for 6 h in tumor-bearing rats. The appetite-stimulating effect of rikkunshito was blocked by the ghrelin receptor antagonist (D-Lys3)-GHRP-6 (2 μmol/kg, i.v.), suggesting that endogenous ghrelin plays a role in rikkunshito’s effects. The frequency of phase III-like contractions in the antrum and duodenum, which is fasting motor activity mediated by ghrelin signaling, decreased in tumor-bearing rats. Rikkunshito (1,000 mg/kg) gradually restored the phase III-like contractions. Additionally rikkunshito (500 mg/kg, p.o. twice daily) prolonged survival in tumor-bearing rats, and this effect was enhanced by the concomitant administration of cisplatin (CDDP; 1 mg/kg, i.p., twice a week from 6 days).

Stomach cancer patients have the highest incidence of cachexia. Terawaki et al. (2014) examined the effects of rikkunshito in a novel stomach cancer cachexia model by implanting nude rats with 85As2 cells. The 85As2 cells line is derived from peritoneal metastasis of the orthotopically implanted human stomach cancer cell line MKN45cl85 and produces LIF, which is a known cachectic factor. This cachexia model involves significant anorexia, weight loss, body composition changes, increased inflammatory marker levels, and low serum albumin levels, fulfilling the cachexia diagnostic criteria. Rikkunshito (orally administered twice daily at 1,000 mg/kg/day for 7 days starting 14 days after the implantation of 85As2 cells in rats) resulted in increased food and water intake rates. Furthermore, rikkunshito substantially alleviated body weight loss and reductions in body compositions, such as fat-free mass, total body water, and total musculature weight, in the 85As2-induced cachexia rat. The anti-cachectic effects of rikkunshito are not related to tumor regression or plasma LIF levels. Therefore, these effects of rikkunshito are likely mediated by activating the GHS-R-NPY/AgRP orexigenic signaling pathway.

Tsubouchi et al. (2014a) examined the impact of rikkunshito on BLM-induced pulmonary fibrosis in mice as a model of pulmonary cachexia. In BLM mice, the administration of rikkunshito (1000 mg/kg, p.o.) for 14 days ameliorated the decrease in body weight and food intake as well as pulmonary inflammation and fibrosis. In BLM-treated *ghrelin^-/-^* and *Ghsr^-/-^* mice, rikkunshito improved pulmonary inflammation, while failing to inhibit the BLM-associated decrease in food intake and body weight (Tsubouchi et al., 2014b). Therefore, the effects of rikkunshito on anorexia and weight loss were assumed to be mediated by ghrelin signaling.

The beneficial effect of rikkunshito on survival was also demonstrated in human patients through a retrospective analysis. Pancreatic cancer patients with ascites (stage III and IV) received gemcitabine or gemcitabine plus rikkunshito. The median survival of pancreatic cancer patients with ascites who were treated with gemcitabine was significantly prolonged by the administration of rikkunshito (Fujitsuka et al., 2011). Future, large-scale clinical trials are required to determine the efficacy and safety of rikkunshito on cancer cachexia.

## CONCLUSION

Cachexia syndrome develops during the advanced stages of various chronic diseases and leads to a decreased QOL and increased rate of morbidity and mortality in patients. The Kampo medicine rikkunshito is prescribed for various upper GI syndromes, such as anorexia, and is very important in the treatment of cachexia-associated weight loss. Clinical and basic studies demonstrate that rikkunshito ameliorates anorexia and cachexia, which may be mediated by synergistically promoting endogenous ghrelin activity by several components of rikkunshito. The use of a ghrelin potentiator, such as rikkunshito, is expected to represent a novel approach for the treatment of anorexia–cachexia syndrome, which is characterized as a decrease in ghrelin signaling with both ghrelin insufficiency and resistance.

## Conflict of Interest Statement

The Guest Associate Editor Akio Inui declares that, despite having collaborated with authors Naoki Fujitsuka and Yasuhito Uezono, the review process was handled objectively and no conflict of interest exists. Yasuhito Uezono has received grant support from Tsumura & Co. Naoki Fujitsuka is employed by Tsumura & Co.
